# Enhanced Biosensor Platforms for Detecting the Atherosclerotic Biomarker VCAM1 Based on Bioconjugation with Uniformly Oriented VCAM1-Targeting Nanobodies

**DOI:** 10.3390/bios6030034

**Published:** 2016-07-05

**Authors:** Duy Tien Ta, Wanda Guedens, Tom Vranken, Katrijn Vanschoenbeek, Erik Steen Redeker, Luc Michiels, Peter Adriaensens

**Affiliations:** 1 Biomolecule Design Group, Institute for Materials Research (IMO), Hasselt University, Diepenbeek BE-3590, Belgium; duytien.ta@uhasselt.be or tdtien@ctuet.edu.vn (D.T.T.); wanda.guedens@uhasselt.be (W.G.); tom.vranken@uhasselt.be (T.V.); 2 Faculty of Food Technology and Biotechnology, Can Tho University of Technology, Can Tho 900000, Vietnam; 3 Immunology and Biochemistry, Biomedical Research Institute (Biomed) and School of Life Sciences, Transnationale Universiteit Limburg, Hasselt University, Diepenbeek BE-3590, Belgium; katrijn.vanschoenbeek@uhasselt.be (K.V.); luc.michiels@uhasselt.be (L.M.); 4 Maastricht Science Programme, Maastricht University, Maastricht 6200 MD, The Netherlands; erik.steenredeker@maastrichtuniversity.nl; 5 Applied and Analytical Chemistry, Institute for Materials Research (IMO), Hasselt University, Diepenbeek BE-3590, Belgium

**Keywords:** uniformly oriented bioconjugation, biosensor, CuAAC, expressed protein ligation, VCAM1-targeting nanobody

## Abstract

Surface bioconjugation of biomolecules has gained enormous attention for developing advanced biomaterials including biosensors. While conventional immobilization (by physisorption or covalent couplings using the functional groups of the endogenous amino acids) usually results in surfaces with low activity, reproducibility and reusability, the application of methods that allow for a covalent and uniformly oriented coupling can circumvent these limitations. In this study, the nanobody targeting Vascular Cell Adhesion Molecule-1 (NbVCAM1), an atherosclerotic biomarker, is engineered with a *C*-terminal alkyne function via Expressed Protein Ligation (EPL). Conjugation of this nanobody to azidified silicon wafers and Biacore™ C1 sensor chips is achieved via Copper(I)-catalyzed azide-alkyne cycloaddition (CuAAC) “click” chemistry to detect VCAM1 binding via ellipsometry and surface plasmon resonance (SPR), respectively. The resulting surfaces, covered with uniformly oriented nanobodies, clearly show an increased antigen binding affinity, sensitivity, detection limit, quantitation limit and reusability as compared to surfaces prepared by random conjugation. These findings demonstrate the added value of a combined EPL and CuAAC approach as it results in strong control over the surface orientation of the nanobodies and an improved detecting power of their targets—a must for the development of advanced miniaturized, multi-biomarker biosensor platforms.

## 1. Introduction

In the recent years, innovative biosensor developments have made several applications with rapid detection and high accuracy possible for real-time environmental monitoring, disease diagnostics and therapy [1,2,3,4,5,6,7]. During the last decade, protein-based—or more particularly antibody-based—biosensors are considered as the most commonly developed platforms [8,9]. Regarding the implementation of antibody candidates in biosensor design, the variable domain of the heavy chain of the single-domain antibody (VHH)—or nanobody—found in camelidae has dedicated a great potential due to numerous advantages over the conventional antibodies [10]: small nanometer size, ease of genetic manipulation and expression in *Escherichia coli*, high stability and antigen-binding capacity as compared to full-length antibodies [11].

Many of the available surface bioconjugation methods rely on physico-chemical adsorption of the detecting proteins (also called ligands) onto the surface of the particular solid support [12,13,14,15] due to its simplicity and low cost. However, this approach most frequently results in a weak interaction and non-uniformly oriented (random) deposition of the proteins on the surface [16,17,18,19], leading to rather poor sensitivity, specificity and reproducibility of the application [15,17,20]. The main problems can be found in the limited number of available binding sites due to the heterogeneous orientation of the proteins on the surface, in addition to the leaching out/washing away during the binding and detection phases. Approaches to create more stable protein-conjugated surfaces frequently make use of strong and covalent couplings between functional groups of the endogenous amino acids, e.g., the thiol group of cysteine or the amine group of lysine, and reactive groups on the surface [13,17,19,21,22]. Although being covalently and so stably coupled, the orientation of proteins immobilized in such a way is still heterogeneous since, in most cases, multiple copies of these endogenous functional groups appear in a protein [17,20]. Alternatively, linkages between the protein and the surface can be established in a more controllable way by fusing the protein of interest with an affinity tag, e.g., a polyhistidine by which an interaction with Ni^2+^, Cu^2+^ or Co^2+^ cations can be established [18]. A number of studies have reported the use of Cu(II)-complexing layers deposited on gold electrodes to obtain a uniformly oriented conjugation of His_6_-tagged proteins, e.g., a dipyrromethene-Cu(II) layer and Receptor domains for advanced glycation end products (RAGE) [23,24], a dipyrromethene-Cu(II) layer and antibodies against H5N1 influenza virus [25], a pentetic acid-Cu(II) layer and JAK2 kinase [26], an iminodiacetic acid-like-Cu(II) layer and RIO1 kinase [27]. The thiol derivatives of these complexing reagents are firstly linked to the gold surfaces to form the layers, followed by Cu(II) complexation and His_6_-tagged protein immobilization. Since the detecting method relies on changes in electrochemical properties of the Cu(II) redox centers upon binding of the analytes to the His_6_-tagged proteins, the copper-complexing layers play the role of both an immobilizing mediator and a signal transducer, which is a big advantage in biosensor design. These studies reported a rather generic method for fabrication of electrochemical biosensors. However, the linkage between the His_6_-tag and the transition metal cations can still be disturbed by pH change or competitive presence of imidazole, thus probably resulting in a loss of activity and reproducibility in such cases. Therefore, to combine the best of both worlds, the investigation of a stable uniformly oriented and covalent immobilization strategy is of utmost importance [10].

An alternative promising approach for controlled immobilization can be found in the site-specific introduction of a bio-orthogonal functional group in the protein. By this, a stable covalent and homogenously oriented linkage can be established between the protein and a complementary functionalized substrate surface. Such highly selective couplings can be achieved by means of “click” chemistry reactions like the copper(I)-catalyzed Huisgen 1,3-dipolar azide-alkyne cycloaddition (CuAAC) [28,29,30]. This reaction specifically occurs between an azide and an alkyne moiety to form a stable 5-membered 1,2,3-triazole ring under Cu(I) catalysis and mild reaction conditions. Following such strategies, a protein can be coupled not only stably but also with a uniform orientation to a solid substrate, resulting in highly functional and homogeneously covered surfaces. To site-specifically introduce a bio-orthogonal group such as alkyne or azide in a protein, the Expressed Protein Ligation (EPL) technique—first described by Muir et al. [31]—has recently (re)gained significant interest as a promising strategy [19,32,33,34,35,36]. Whereas EPL on the one hand has been employed for the site-specific alkynation of nanobodies and “click” chemistry and on the other hand for the selective coupling of proteins to micelles and polymersomes [37,38,39,40,41,42], studies on using the combined EPL and “click” chemistry approach to create nanobody-based biosensor platforms for detecting atherosclerosis have not been reported.

Recently, we developed an efficient protocol to functionalize nanobodies targeting the Vascular Cell Adhesion Molecule-1 (abbreviated as NbVCAM1) at the *C*-terminus with an alkyne function in order to be coupled to azide-functionalized supports with a uniform orientation via CuAAC chemistry [43]. VCAM1 is one of the inducible adhesion molecules that play a role in mediating the recruitment and attachment of leukocytes to the vascular endothelium [44] as well as further migration into the subendothelial spaces [45]. These are the major processes in the development of atherosclerotic plaques [46]. The protein is also unraveled to be associated with other diseases including rheumatoid arthritis [47], inflammatory bowel disease [48], multiple sclerosis [49], renal cell carcinoma [50], renal tumors [51] and colorectal cancer [52,53]. Therefore, VCAM1 might be considered as a pre-diagnostic marker and promising drug target for cardiovascular diseases and cancer diagnosis/therapy follow-up [54]. This explains the selection of the NbVCAM1 to demonstrate the proof of principle of the proposed conjugation strategy for the development of improved label-free optical biosensors. In particular, the *C*-terminally alkynated nanobodies are conjugated by CuAAC “click” chemistry to diverse azide-functionalized solid surfaces, including silicon wafers and gold-coated Biacore™ sensor chips, for VCAM1 antigen binding detection via ellipsometry or SPR, respectively. The resulting nanobody-conjugated surfaces display significant improvements in antigen binding capacity/affinity, sensitivity, detection limit, quantitation limit and reusability as compared to those produced via random coupling methods. Furthermore, the approach is generic and can be easily translated to other proteins.

## 2. Materials and Methods 

All chemicals and reagents were purchased from Sigma unless stated otherwise. The PCR reagents, restriction enzymes and B-PER reagent were obtained from Thermo Scientific. The cysteine-alkyne bifunctional linker (Figure S1A, Supplementary Materials) was purchased from Eurogentec, the azido-propylamine linker from Jena bioscience (Figure S1B, Supplementary Materials) and the *N*-hydroxysuccinimide (NHS) derived ester linker 2,5-dioxopyrrolidin-1-ylhex-5-ynoate (Figure S1C, Supplementary Materials) was self-synthesized according to Jagadish et al. [55]. The pMXB10 vector, *E. coli* SHuffle^®^ T7 competent cells and chitin resin were purchased from New England Biolabs. The two recombinant human VCAM1 antigens (hVCAM1) were bought from R & D Systems (MW of 270 kDa) and Peprotech (MW of 180 kDa) while the recombinant mouse VCAM1 (mVCAM1) antigen was purchased from Bioconnect (Huissen, The Netherlands, MW of 95 kDa). The ellipsometer and silicon wafers were bought from Synapse B.V. (Maastricht, The Netherlands) and the Biacore™ (Diegem, Belgium) C1 sensor chips from GEHealthcare. The SPR experiments were performed with a Biacore™ T200 model (GE Healthcare).

### 2.1. Preparation of the Nanobody Variants

The NbVCAM1-LEY nanobody was expressed as a chimeric protein (fusion with an intein and a chitin binding domain) in the *E. coli* SHuffle^®^ T7 strain and was subjected to EPL-mediated cleavage with DTT (to produce NbVCAM1-LEY) or with the cysteine-alkyne linker to produce the *C*-terminally alkynated NbVCAM1-LEY, i.e., NbVCAM1-LEY-alkyne, as previously described [13]. The non-VCAM1-targeting NbBcII-10-LEY-alkyne (an anti-bacterial β-lactamase nanobody) was produced using the same protocol and used as a non-binding reference (negative control) in ELISA and SPR experiments. Details of expression and purification of these nanobodies are provided in the Supplementary Materials. The NbVCAM1-His_6_ was produced as described by Saerens et al. [56] and used as an antigen-binding reference (a positive control in ELISA and a randomly conjugated reference via EDC/NHS chemistry in SPR). Random alkynation of the NbVCAM1-His_6_ was performed for 3 h in 100 μL PBS buffer (137 mM NaCl, 2.7 mM KCl, 10 mM Na_2_HPO_4_ and 2 mM KH_2_PO_4_ at pH 7.4) containing 20 μM nanobody and a 10-fold molar excess of the *N*-hydroxysuccinimide (NHS) derived ester linker 2,5-dioxopyrrolidin-1-ylhex-5-ynoate. This linker carries a terminal alkyne function and its coupling to the nanobody can be accomplished via the reaction between the free and accessible amino groups of the protein (*N*-terminal NH_2_ and lysine ε-NH_2_) and the linker’s carbonyl carbon, resulting in a stable amide bond. The resulting protein isomer mixture (designated as rNbVCAM1-His_6_-alkyne with “r” denoting “randomly”) was then immediately filtered through a Zeba™ Spin Desalting Column, 7K MWCO (Thermo Scientific, Aalst, Belgium) and used as a reference for random coupling in the ellipsometry and SPR experiments. Protein concentrations were determined with the BCA Protein Assay kit (Thermo Scientific).

### 2.2. Characterization of the NbVCAM1-LEY-alkyne by Mass Spectrometry, Western Blotting and ELISA

To test the: (i) purity; (ii) level of *C*-terminal alkynation; and (iii) “clickability” of the NbVCAM1-LEY-alkyne before use in subsequent coupling experiments, the NbVCAM1-LEY-alkyne was subjected to ElectroSpray Ionization-Fourier Transform Mass Spectrometry (ESI-FTMS), CuAAC-mediated biotinylation and ELISA, as described in the Supplementary Materials.

### 2.3. Azidification of the Support Surfaces for Ellipsometry and SPR

For ellipsometry, finely cut silicon wafers (0.3 × 3.2 cm) were first cleaned in a 3:1 mixture (*v*/*v*) of H_2_SO_4_:H_2_O_2_ for 5 min, followed by treatment with 6% (*v*/*v*) HF for a few seconds to create a hydrophobic surface. The slides were then oxidized with chromic acid (8% potassium dichromate (*w*/*v*) in 25% H_2_SO_4_ (*v*/*v*)) at 80 °C for 1 h to make them hydrophilic. The oxidized slides were washed with MilliQ water and ethanol before silanization with a solution of 15% *N*-(trimethoxysilylpropyl)ethylenediamine-triacetic acid-trisodium salt in 0.2 M acetate buffer for 1 h at 100 °C. In this way, the slides were carboxylated and after washing with MilliQ water and ethanol, the slides were azidified by submerging in a functionalization solution for 3 h at room temperature in the dark. The functionalization solution is composed of 0.4 M *N*-(3-dimethylaminopropyl)-*N*′-ethylcarbodiimide hydrochloride (EDC), 0.3 M *N*-hydroxysuccinimide (NHS) and 1 mM azido-propylamine in 220 mM HEPES buffer pH 6.8. The slides were then blocked in 1 M aminoethoxyethanol pH 7.8 for 45 min before washing with MilliQ and ethanol. The functionalized slides were N_2_-dried and kept in the dark at room temperature if not used immediately.

For SPR, the commercial Biacore™ Series S carboxymethylated C1 chips were washed five times with PBS running buffer (10 μL/min) before azidification. All stock solutions were prepared in Biacore compatible plastic vials according to the manufacturer instructions (e.g., 75 mg/mL EDC, 11.5 mg/mL NHS and 15 mM azido-propylamine). The functionalization process was performed automatically according the preset EDC/NHS coupling protocol of the T200 Biacore™ Control software. The first three flow cells (Fc) of the chip were functionalized with the azido-propylamine linker (for coupling to alkynated nanobodies later via CuAAC chemistry), whereas Fc4 was directly conjugated with NbVCAM1-His_6_ (10 μM) via a peptide bond between the COOH groups of the chip and an NH_2_ group of the nanobody. The chips were than washed 5 times with PBS and blocked by flushing with 1 M aminoethoxyethanol pH 7.8 at a flow-rate of 10 μL/min for 10 min. The azidified chips (except for Fc4) were then washed 5 times with PBS and stored in PBS at 4 °C if not used immediately.

### 2.4. Ellipsometry

*pH optimization for CuAAC-mediated conjugation*. Ellipsometry was performed in null-mode [57] on a semi-automatic ellipsometer with a built-in control software and the following settings: filter 16; offset 0; gain 1; polarizer sweep angle 140° and analyzer sweep angle 175°. The functionalized silicon slides were assembled in a sample holder with eight positions and an area of approximately 0.09 cm^2^ (0.3 × 0.3 cm) was dipped in 400 μL PBS in quartz cuvettes, followed by a baseline stabilization during 5 min. An external incubator is used for subsequent washing steps. Coupling with different nanobody variants was performed by either physisorption or CuAAC “click” chemistry. The CuAAC-mediated coupling was first carried out on azidified surfaces in different buffers (PBS and 10 mM sodium acetate at pH 4.0, 5.0 or 6.0) in order to select the most efficient pH. Hereto, the coupling cocktail was prepared in a quartz cuvette containing 400 μL mixture of 1 μM nanobody (NbVCAM1-LEY, NbVACM1-LEY-alkyne, rNbVCAM1-His_6_-alkyne or NbVCAM1-His_6_), 2.5 mM sodium l-ascorbate, 1 mM THPTA (Tris(3-hydroxypropyltriazolylmethyl)amine) and 0.5 mM CuSO_4_. To prove the selectivity of the “click” reaction, two types of silicon substrates, i.e., carboxylated and azidified slides, were evaluated in acetate buffer pH 4.0. The Cu(II) salt is reduced to the Cu(I) catalyst by sodium ascorbate as a reducing reagent. Due to its high instability, Cu(I) is stabilized by THPTA complexing ligand [25]. The CuAAC coupling reaction was performed for 30 min at room temperature followed by consecutive washing steps with washing buffer (WB) (200 mM dihydrogen sodium phosphate, 200 mM sodium chloride, 150 mM ethylenediaminetetraacetic acid, and 50 mM ethanolamine at pH 7.5); 0.5% (*w*/*v*) SDS; PBS; 0.7 M β-mercaptoethanol (BME); and PBS (5 min for each washing step). For all nanobody conjugation experiments, the amount of surface coverage was quantified during the first PBS washing step on the basis of the sweep angle shift of the polarizer ΔP to maintain the nullified status of the reflected light on the silicon surfaces. This angle shift is proportional to the difference in surface mass density before conjugation (baseline) and during the first PBS washing. The surface mass density (Γ, ng protein/cm^2^) can be calculated using the simplified Lorentz–Lorenz relation Γ = 85 × ΔP [58,59]. The reported results represent the average of four measurements.

*Nanobody conjugation and antigen binding*. The NbVCAM1-LEY-alkyne and rNbVCAM1-His_6_-alkyne were conjugated to the azidified slides by CuAAC as described above. A random physisorption reference was performed with non-alkynated NbVCAM1-LEY on a carboxylated slide. For this, the slide was incubated for 30 min with 1 μM nanobody in 400 μL TBS buffer pH 7.5 (50 mM Tris and 0.1 M NaCl), followed by washing with PBS to remove only the unbound proteins but not the adsorbed nanobodies. The recombinant hVCAM1 antigen (R & D Systems) was prepared in PBS at a stock concentration of 100 μg/mL (0.37 μM). In order to compare the maximal antigen binding capacity of the nanobody conjugated silicon wafers, the stock solution was only diluted to a final concentration of 5 μg/mL in 400 μL PBS and added to the cuvettes containing the slides conjugated with NbVCAM1-LEY-alkyne or rNbVCAM1-His_6_-alkyne (via CuAAC), or NbVCAM1-LEY (by physisorption). The antigen–nanobody association was performed for 30–50 min under stirring until saturation was reached. The slides were then consecutively washed with WB, glycine-HCl buffer (0.1 M glycine-HCl and 0.2 M NaCl at pH 2.5), SDS and PBS. The ΔP and Γ values were determined by averaging the data points measured during the washing with WB. To test the reusability of the nanobody-conjugated surfaces, the slides were subjected to a second antigen binding following the same binding protocol as described above. All antigen binding measurements were performed in duplicate.

*Determination of the dissociation constant (K_D_) of the antigen binding*. The hVCAM1 binding to surfaces conjugated with NbVCAM1-LEY-alkyne or rNbVCAM1-His_6_-alkyne was evaluated for increasing initial antigen concentrations (0, 1.25, 2.5, 3.75 and 5 μg/mL in PBS) until saturation was achieved. The surfaces were then washed with WB and PBS. The affinity constant is determined using the Scatchard equation:

(1) [hVCAM1bound][hVCAM1free]=n×C×1KD−[hVCAM1bound]×1KD

in which [hVCAM1_bound_] is the concentration (nM) of bound hVCAM1 at equilibrium as determined during the PBS washing, n is the number of binding sites per molecule (assumed to be 1 if monovalent), and C is the total amount of binding sites available. The [hVCAM1_bound_] is proportional to the increase of the surface mass density increase (Γ) at equilibrium and the contact surface area (0.09 cm^2^), and inversely proportional to the reaction volume (V, 400 μL or 4 × 10^−4^ L), and the MW (270,000 Da) of the hVCAM1 (R & D Systems), and can be calculated as:

(2) $$\left[{\text{hVCAM1}}_{\text{bound}}\right]=\frac{\Gamma \times 0.09}{270,000\times 4\times 4^{-4}}$$

For each initial antigen concentration [hVCAM1_0_], the free hVCAM1 concentration can be determined according to:

[hVCAM1_free_] = [hVCAM1_0_] − [hVCAM1_bound_] (3)

The Scatchard plots were made using the data obtained from Equations (1)–(3) and a linear regression of the [hVCAM1_bound_]/[hVCAM1_free_] ratio as a function of the [hVCAM1_bound_] was performed in order to derive the K_D_ (nM).

*Determination of antigen binding sensitivity.* For increasing [hVCAM1_0_], the equilibrium amount of hVCAM1 bound to the surface conjugated with NbVCAM1-LEY-alkyne or rNbVCAM1-His_6_-alkyne was used to construct the dose–response curves. Hereto, the surface mass densities were plotted against the corresponding antigen concentrations and a linear least squares fit (95% confidence level) was carried out using GraphPad Prism 5.0 software. The slopes of the curves were also used as a criterion to compare the antigen detection sensitivity according to Thevenot et al. [60].

*Determination of detection limit (DL) and quantitation limit (QL)*. DL is defined as the lowest concentration of an analyte in a sample that can be detected. It can be determined as DL = 3.3 × σ/S where S and σ are the slope and the standard deviation of the intercept, both of which are obtained from the dose–response curve. QL is defined as the lowest concentration of an analyte in a sample that can be quantitated and can be determined as QL = 10 × σ/S [61].

### 2.5. Surface Plasmon Resonance

*Nanobody conjugation*. The azidified C1 chip (described before) was first washed five times with PBS to obtain a stable baseline. The first three flow cells were then conjugated via CuAAC with 10 μM of NbBcII-10-LEY-alkyne (Fc1), NbVCAM1-LEY-alkyne (Fc2), and rNbVCAM1-His_6_-alkyne (Fc3) using the “click” cocktail in acetate buffer pH 4.0 as described for ellipsometry earlier (the Fc4 was already conjugated with 10 μM NbVCAM1-His_6_ via EDC/NHS coupling). The “click” cocktail was injected at a flow-rate of 10 μL/min for 30 min. After that, the chip was washed five times and stored in PBS at 4 °C if not used immediately. The immobilization levels, expressed in Response Units (RU), were determined 10 s before the end of the PBS washing step (Figure S2, Supplementary Materials).

*Analysis of the binding kinetics*. Three antigens were employed in this study: two hVCAM1 antigens (270 kDa and 180 kDa from R & D Systems and Peprotech, respectively), and mVCAM1 (95 kDa from Bioconnect). The antigens were injected using a default single-cycle kinetics protocol [62], i.e., a series of initial PBS injections without antigen, followed by a continuous injection of antigen at increasing concentration between 0–1000 ng/mL in PBS. For each concentration, the association and dissociation steps were carried out during respectively 10 min (30 μL/min) and 2 min (30 μL/min), after which a final dissociation step of 10 min (30 μL/min) was accomplished. The sensorgrams of Fc2, 3 and 4 were double referenced. This includes subtraction of blanks (PBS only) in addition to the contributions of the reference Fc1 (i.e., [FcX − Fc1]_antigen_ − [FcX − Fc1]_PBS_ where X is 2, 3, 4). The binding kinetic parameters (k_a_ and k_d_) and the dissociation constant (K_D_) were determined from fits using the 1:1 binding model implemented in the T200 BIAevaluation software. Regeneration of the chip was performed by injecting 10 mM NaOH (Table S4, Supplementary Materials) at a flow-rate of 100 μL/min for 1 min before starting the next kinetic binding run. 

*Determination of antigen binding sensitivity, detection limit and quantitation limit*. The dose–response curves for hVCAM1 binding to the conjugated nanobodies on Fc2, 3 and 4 for different hVCAM1 concentrations were constructed by the BIAevaluation software. The sensitivity of antigen detection was calculated based on the slopes of the linear curves. The DL and QL are determined as described for ellipsometry. 

## 3. Results and Discussions

### 3.1. Expression and EPL-Mediated Alkynation of NbVCAM1-LEY

**Figure 1:**
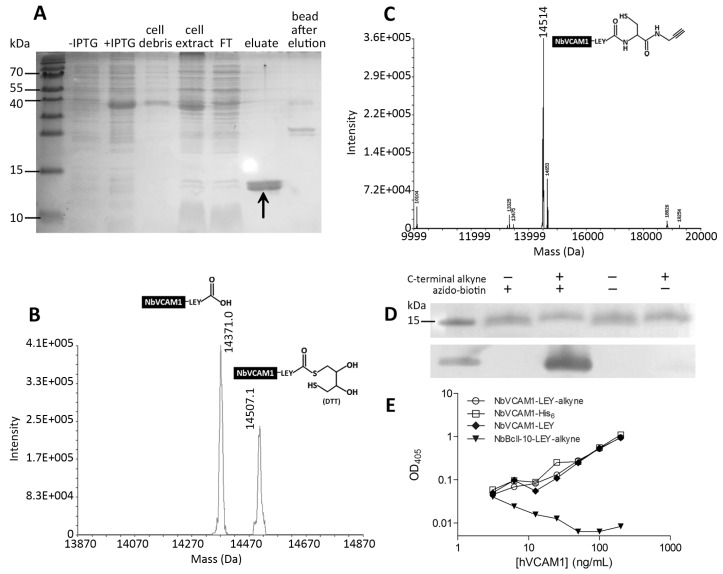
(**A**) SDS-PAGE of cytoplasmic expression and purification steps of NbVCAM1-LEY-alkyne during the EPL process. From left to right: non-induced and Isopropyl β-D-1-thiogalactopyranoside--induced total cell proteins, the cell debris and lysate, the proteins not bounded to the chitin column (FT: flow through), the eluted fraction (alkynated NbVCAM1-LEY) (arrow) and the extract of the beads after EPL; (**B**,**C**) Mass spectra of the purified NbVCAM1-LEY and NbVCAM1-LEY-alkyne fractions, respectively, with their structures; (**D**) CuAAC-mediated biotinylation of NbVCAM1-LEY-alkyne and analysis by SDS-PAGE (top) and Western blot (bottom), demonstrating the presence of the alkyne “click” function; (**E**) Sandwich ELISA to show the hVCAM1 antigen binding capacity of different nanobody variants. The experiment was performed in triplicate and the data are plotted on a log-log scale. The non-alkynated, fully active NbVCAM1-His_6_ and the NbBcII-10-LEY-alkyne (will not bind VCAM1—see Supplementary Materials for more details about the production of these nanobodies) are used as positive and negative controls, respectively.

In this study, non-alkynated nanobodies (NbVCAM1-LEY) and *C*-terminally alkynated nanobodies (NbVCAM1-LEY-alkyne) were produced, purified and desalted according to a previously optimized EPL-assisted protocol [38] (see Supplementary Materials for more details). The NbVCAM1-LEY-alkyne was obtained in good yield (~20 mg per liter of culture) and high purity (Figure 1A). Characterization of these nanobodies was performed with ESI-FTMS. The non-alkynated protein fraction (resulting from the intein cleavage with dithiothreitol (DTT)-containing buffer during EPL process) shows two masses: one of NbVCAM1-LEY-DTT and one of the hydrolyzed NbVCAM1-LEY (Figure 1B). This fraction was referred to as “NbVCAM1-LEY” in further experiments. The mass spectrum of the NbVCAM1-LEY-alkyne is shown in Figure 1C and the presence of an alkyne “click” function was confirmed by a successful coupling to an azido-biotin derivative (Figure S1D, Supplementary Materials) followed by Western blotting (Figure 1D). The *C*-terminally alkynated nanobody retained a similar binding capacity towards the hVCAM1 antigen as the reference non-alkynated NbVCAM1-His_6_ in an ELISA test (Figure 1E). This procedure allows a generic preparation of *C*-terminally alkynated nanobodies as starting materials for CuAAC-mediated bioconjugation.

### 3.2. Buffer Selection for Optimal CuAAC-Mediated Surface Conjugation

The efficiency of the CuAAC-mediated coupling of monoalkynated NbVCAM1-LEY to azidified silicon wafers was evaluated for different buffers. As monitored by ellipsometry, the highest amount of nanobody conjugation was found for acetate buffer pH 4.0 (Table S1, Supplementary Materials). Therefore, this buffer was selected for all further CuAAC-mediated couplings of alkynated nanobodies to the silicon surfaces and the Biacore™ C1 sensor chips. 

**Figure 2:**
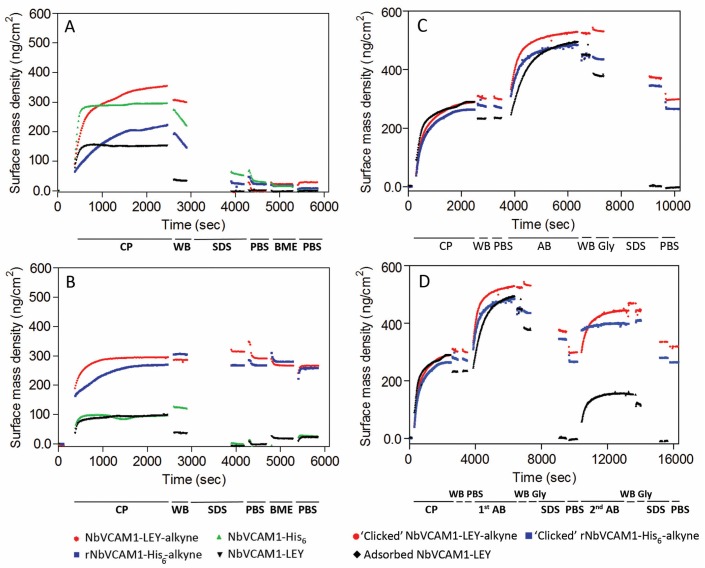
Ellipsometric results for the CuAAC-mediated coupling of different NbVCAM1 variants in acetate buffer pH 4.0 to: (**A**) carboxylated; and (**B**) azidified silicon wafers. The coupling process (CP) was followed by consecutive washing steps with washing buffer (WB), SDS, phosphate buffer saline (PBS), BME and PBS. Data were recorded in real-time and in quadruplicate and one representative curve is presented; (**C**) A one-cycle human VCAM1 antigen (R & D Systems) binding (A,B) on different nanobody-conjugated silicon surfaces. The nanobodies were immobilized using either CuAAC (for the rNbVCAM1-His_6_-alkyne and NbVCAM1-LEY-alkyne) or physisorption (for the NbVCAM1-LEY). Binding was followed by consecutive washing steps with WB, Glycine-HCl, SDS and PBS; (**D**) A two-cycle AB binding process on similar slides. Measurements were carried out in duplicate and one representative curve is shown.

Figure 2A,B present ellipsometric data that clearly demonstrate the covalent clicking of the alkynated nanobodies to the azidified silicon slides but not to the carboxylated slides for which all nanobodies were removed by washing with washing buffer (WB) and SDS solution. Coupling of rNbVCAM1-His_6_-alkyne (also known as randomly alkynated nanobody variant, see experimental section for synthesis details) and NbVCAM1-LEY-alkyne to azidified slides on the other hand results in an increase of the average surface mass density by respectively 267 and 291 ng protein/cm^2^ (Figure 2B). This is in contrast to non-alkynated nanobodies (NbVCAM1-His_6_ and NbVCAM1-LEY), which were washed away by the WB and SDS solution (Figure 2B).

### 3.3. Study of the Antigen Binding by Ellipsometry

The antigen binding capacity of various nanobody-conjugated silicon wafers was examined by adding an excess of hVCAM1 antigen (5 μg/mL) in order to allow a maximum, saturating antigen binding (Figure 2C). Since the NbVCAM1-His_6_ contains five amine groups (4 lysine ε-NH_2_ and 1 α-NH_2_) that can react with the NHS-alkyne linker (see Experimental Section and Supplementary Materials for more details), the resulting rNbVCAM1-His_6_-alkyne can theoretically be coupled to the azidified surface in five different orientations. Likewise, the physically adsorbed NbVCAM1-LEY is also coupled to the surface with random orientations. In contrast, the NbVCAM1-LEY-alkyne is covalently coupled with a unique orientation to the azidified surface and shows a larger surface density increase as compared to the others. This demonstrates that more binding sites are available for the target if the surface is conjugated with uniformly oriented nanobodies. Moreover, because the *N*-terminus of the nanobody is importantly involved in antigen recognition [63,64,65,66], coupling via the *N*-terminal amine function will for sure be harmful for the activity. If the nanobodies are covalently coupled via the alkynated *C*-terminus on the other hand, all will have their active sites available for the antigen binding.

In order to explore the repeatability of the antigen binding for recycling the biosensing platforms, the antigen-loaded surfaces were washed thoroughly (successively with WB, Glycine-HCl, SDS, and PBS) to remove all bound antigen, after which the slides were subjected to a second cycle of antigen binding (Figure 2D). For the CuAAC conjugated surfaces, there was no significant difference in surface mass density after these washing steps as compared to the situation before the first antigen binding cycle, indicating that the covalent linkage between the alkynated nanobodies and the azidified surfaces is stable and can resist the regeneration process. In case of physically adsorbed nanobodies on the other hand, both the antigens and the nanobodies were washed away during the SDS washing. This nicely demonstrates the improvement in stability of the NbVCAM1-LEY-alkyne conjugated via CuAAC as compared to physisorption. It is remarked that the antigen binding capacity was slightly lower in the second binding cycle, probably due to partial nanobody denaturation by the SDS washing step at the end of the first cycle (Table S2, Supplementary Materials). In spite of this, the second binding cycle confirms the improved antigen binding capacity of the site-specifically conjugated NbVCAM1-LEY-alkyne.

**Figure 3:**
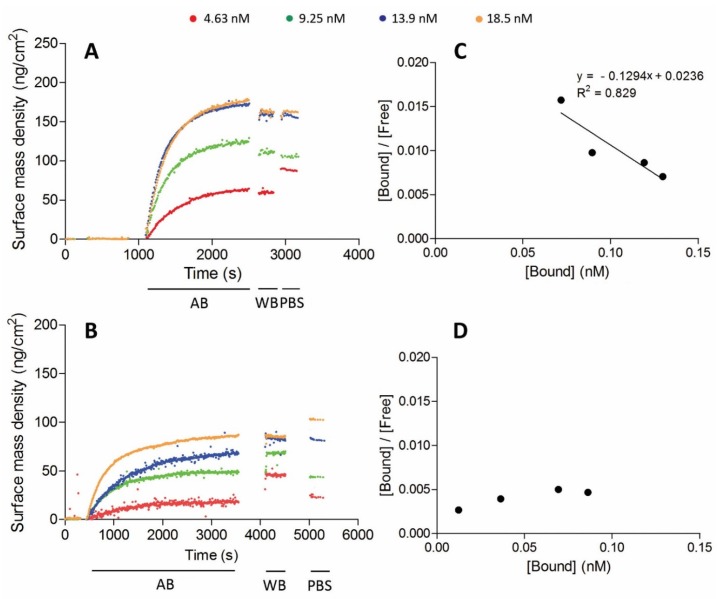
Antigen binding curves of azidified silicon wafers conjugated by CuAAC with: NbVCAM1-LEY-alkyne (**A**); and rNbVCAM1-His_6_-alkyne (**B**) for different hVCAM1 antigen concentrations as detected by ellipsometry. The association process was followed by washing with washing buffer and PBS, after which the surface mass density was determined. (**C**,**D**) The corresponding Scatchard plots. The dissociation constants K_D_ were derived from the inverse of the slopes of the linear regression equations.

The dissociation constant of the antigen binding can be determined from the ellipsometric binding curves (Figure 3A,B). There was a faster hVCAM1 binding of the conjugated NbVCAM1-LEY-alkyne, representing a larger association constant (k_a_), as compared to the conjugated rNbVCAM1-His_6_-alkyne, i.e., 25 vs. 50 min, respectively (after baseline stabilization) to reach saturation. Based on the surface mass density at equilibrium, the Scatchard plots (Figure 3C,D) were constructed from which a K_D_ value of 7.7 nM was determined for the NbVCAM1-LEY-alkyne-conjugated surface. The antigen binding at low concentration of the rNbVCAM1-His_6_-alkyne-conjugated surface is so poor that a very large but unreliable K_D_ is resulted from the Scatchard plot. However, it confirms the increase in binding affinity of the site-specifically alkynated nanobody after surface conjugation. These results indicate that there are not only more binding sites available on the surfaces conjugated with the oriented NbVCAM1-LEY-alkyne, but also that these sites have an equal binding affinity towards the hVCAM1 antigen. In contrast, a rNbVCAM1-His_6_-alkyne-conjugated surface has less binding sites and some of them bind the antigen with less affinity due to a conformational change resulting from conjugating via an alkyne which is localized within or close to the binding pocket.

**Figure 4:**
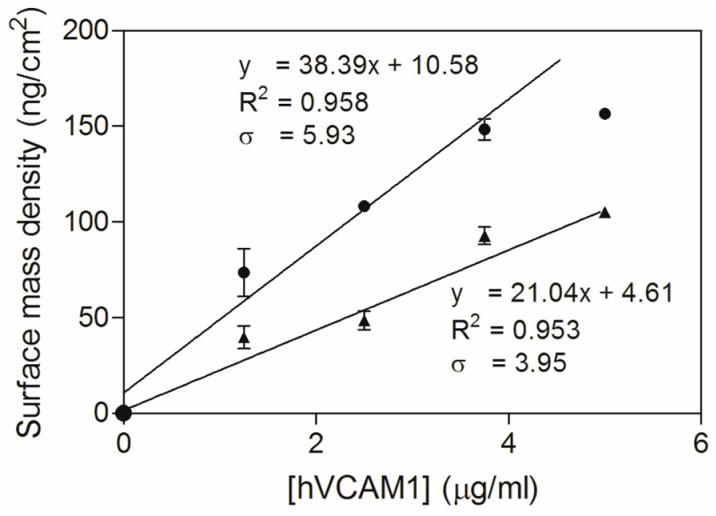
Dose–response curves for the hVCAM1 (R & D Systems) binding to NbVCAM1-LEY-alkyne (●) and rNbVCAM1-His_6_-alkyne (▲) conjugated silicon slides obtained by ellipsometry. The data were analyzed by means of a linear least squares fitting (95% confidence level; note that only the non-saturating antigen concentrations (0–3.75 μg/mL) were taken into account for NbVCAM1-LEY-alkyne conjugated surfaces).

Additionally, the dose–response curves, representing a linear correlation between the surface mass density and the antigen concentration (Figure 4), can be used for comparing antigen binding sensitivity. The slopes of the curves indicate that the sensitivity could be increased 1.82 times (38.39 vs. 21.04) by using the *C*-terminally alkynated nanobody as compared to the randomly alkynated nanobody. It is noticed that the hVCAM1 binding of the surface conjugated with the NbVCAM1-LEY-alkyne reaches saturation at 3.75 μg/mL antigen. Therefore, the dose–response curve was fitted with only the data points in the linear range. The LD and LQ of the NbVCAM1-LEY-alkyne conjugated surfaces are, respectively, 0.51 and 1.54 μg/mL, and so slightly lower than the values of 0.62 and 1.88 μg/mL found for the rNbVCAM1-His_6_-alkyne conjugated surfaces. It further is experimentally observed that hVCAM1 concentrations below 1.25 μg/mL indeed become difficult to quantify with ellipsometry. In an attempt to achieve a higher sensitivity (i.e., a lower detection limit) for biosensing applications, surface plasmon resonance (SPR) was employed using a Biacore™ T200 workstation.

### 3.4. Study of the Antigen Binding by Surface Plasmon Resonance

Compared to ellipsometry, SPR is a more advanced label-free technique to study biomolecular interactions due to its higher degree of automation and sensitivity in addition to limited sample preparation. In a previous study on hVCAM1 antigen binding using SPR, Broisat et al. [63] reported a dissociation constant in the nanomolar range for NbVCAM1-His_6_. However, these authors immobilized the antigen as the “ligand” and used the nanobody as the “analyte”, a set-up not aimed for bio-sensing applications. In this study, we construct Biacore™ C1 chip surfaces conjugated with nanobodies that are not only bound covalently but also with a uniformly orientation, and therefore exhibit an improved binding sensitivity as compared to randomly bound nanobodies.

**Figure 5:**
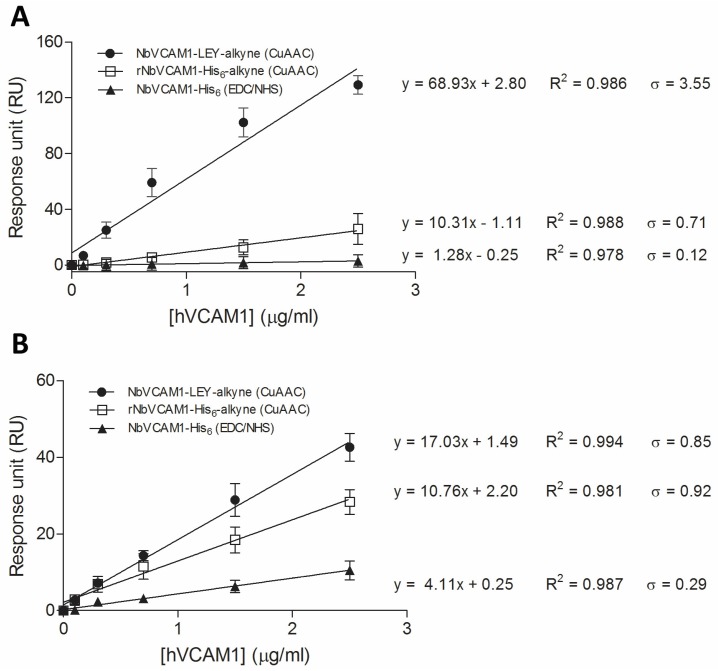
Dose–response curves for the binding of hVCAM1 from: R & D Systems (**A**); and Peprotech (**B**) to VCAM1-targeting nanobodies that are conjugated to different flow cells of the C1 chips via different chemistries.

The improvement in binding sensitivity of conjugated NbVCAM1-LEY-alkyne (as compared to rNbVCAM1-His_6_-alkyne conjugated via CuAAC and NbVCAM1-His_6_ coupled via EDC/NHS chemistry) can be observed for two different hVCAM1 antigens by comparing the slopes of the dose–response curves (Figure 5A,B and Table 1). For the hVCAM1 of R & D Systems, the sensitivity was increased 6.7 times and 53.9 times, respectively. For the hVCAM1 from Peprotech, the sensitivity was increased 1.6 times and 4.1 times, respectively. Improvements in detection and quantitation limit were also observed for the binding of both hVCAM1 antigens to NbVCAM1-LEY-alkyne as compared to the other nanobody variants (Table 1). In addition, and this for ellipsometry as well as for SPR, the antigen binding experiments were performed on three independent surfaces (three replicates) and the resulting measurements show small relative standard deviations and high correlation coefficients (Figure 5), indicative for a good reproducibility.

As shown in Table 2, the binding kinetics and affinity of VCAM1 for the nanobody-conjugated surfaces was further evaluated on the basis of the K_D_ constants. The association constant (k_a_) and dissociation constant (k_d_) are shown in Table S3 (Supplementary Materials). It is firstly noticed that the NbVCAM1-LEY-alkyne conjugated flow cell (Fc) is able to bind both human and mouse VCAM1 antigens with nanomolar affinities, which is in accordance with the findings of Broisat et al. [63]. Moreover, the site-specifically conjugated NbVCAM1-LEY-alkyne shows a significant increase in binding affinity for the two hVCAM1 antigens, i.e., with a factor of 6–14 as compared to rNbVCAM1-His_6_-alkyne and even a factor of 138 as compared to NbVCAM1-His_6_ coupled via EDC/NHS chemistry (Table 2). These data are in accordance with the ellipsometry results, supporting the hypothesis that random conjugation via the lysines (or alkynated lysines) disturbs the conformation of the nanobody’s binding domains if the anchoring amine is directly involved in the antigen binding or is changing the spatial structure of the binding pocket. These results also show that SPR is more sensitive to detect the differences in binding affinity as compared to ellipsometry, mainly due to the lower detection limit (subnanomolar concentrations of antigen) and higher level of automation (limited sample preparation and handling) of the Biacore™ system.

In addition, the difference in K_D_ for the binding of the different antigens to conjugated NbVCAM1-LEY-alkyne (Table 2) can be explained by the structural differences between these antigens. The single domain hVCAM1 (from Peprotech) and mVCAM1 (from Bioconnect) bind with similar affinity constants, whereas the heterodimeric hVCAM1 (from R & D Systems) exhibits a smaller K_D_, i.e., a higher affinity. Since this antigen contains a VCAM1 and a partial IgG1 domain, both of which belong to the immunoglobulin superclass, they might share some structural similarities which probably enables occasional binding to the nanobody with the IgG1 domain (although with much less affinity as compared to the VCAM1 domain). It therefore has to be noted that the results calculated for the binding of the chimeric hVCAM1 antigen by means of the monovalent binding model have to be considered as useful approximations.

After regeneration of the nanobody-conjugated sensor chips, it was shown that 70% of the binding activity was retained for the NbVCAM1-LEY-alkyne based sensor (Table S4, Supplementary Materials), whereas more than 50% was lost for the rNbVCAM1-His_6_-alkyne and the NbVCAM1-His_6_ based reference sensors (data not shown). It again indicates that nanobodies that are conjugated via the *C*-terminally alkyne to the azidified surfaces resist the regeneration conditions better, and are thus more stable, than the nanobodies on the reference sensors. It should be noted that the reusability of all above platforms might be improved upon applying different regeneration protocols.

All the results presented above show that: (i) the EPL technique is an efficient tool to site-specifically append a bio-orthogonal functional group to a nanobody for (ii) site-specific coupling to surfaces for advanced biosensing. As reported before, our adapted EPL protocol allows a one-step synthesis and purification of *C*-terminally alkynated nanobodies in high yield for subsequent CuAAC-mediated coupling [43]. This is highly advantageous as compared to the two-step procedure (purification of NbVCAM1-His_6_ and its random alkynation separately) used to prepare the randomly alkynated nanobodies. However, more importantly, our findings additionally demonstrate that the combined use of EPL and CuAAC chemistry allows to uniformly conjugate solid supports with nanobodies, resulting in higher binding affinities and sensitivities as well as improved detection limit, quantitation limit and repeatability as compared to non-oriented (random) approaches. More specifically for the NbVCAM1-LEY-alkyne nanobody, a SPR-based VCAM1 biosensor would allow to detect human and mouse VCAM1 antigens at (sub)nanomolar concentrations in atherosclerotic serum.

## 4. Conclusions

The combined use of EPL and highly selective CuAAC “click” chemistry is reported in this study in order to develop biosensor platforms for VCAM1 binding detection with significantly improved performances. The proof of principle of the approach, being generic in nature, is demonstrated in this study for a specific NbVCAM1-LEY-alkyne nanobody and SPR-based read-out platform. More specifically, the EPL principle is applied to engineer the nanobody NbVCAM1-LEY with a site-specific, i.e., *C*-terminal, alkynated linker molecule while maintaining full antigen binding capacity. The alkyne function of this linker allows conjugating the resulting NbVCAM1-LEY-alkyne nanobodies covalently and with a uniform orientation to azidified solid supports via a stable triazole linkage resulting from the CuAAC “click” reaction. This paper also demonstrates the potential, feasibility and benefits of this innovative strategy for biosensor development and can pave the way to further device miniaturization needed for the development of sensors for detecting multiple antigens in a single assay. 

## Figures and Tables

**Table 1:** The sensitivity (S), detection limit (DL) and quantitation limit (QL), all displayed in μg/mL, for the binding of recombinant human VCAM1 antigens to the nanobody-conjugated flow cells prepared by the different coupling methods.

	hVCAM1 (R & D System)			hVCAM1 (Peprotech)		
	S	DL	QL	S	DL	QL
NbVCAM1-LEY-alkyne (CuAAC chemistry)	68.93	0.17	0.51	17.03	0.16	0.50
rNbVCAM1-His_6_-alkyne (CuAAC chemistry)	10.31	0.23	0.69	10.76	0.28	0.86
NbVCAM1-His_6_ (EDC/NHS chemistry)	1.28	0.31	0.94	4.11	0.23	0.71

**Table 2:** The dissociation constant (K_D_ in nM ± standard error) for the binding of the human and mouse recombinant VCAM1 antigens to the nanobody-conjugated flow cells prepared by the different coupling methods.

	hVCAM1 (R & D System)	hVCAM1 (Peprotech)	mVCAM1 (Bioconnect)
NbVCAM1-LEY-alkyne (CuAAC chemistry)	0.15 ± 0.01	1.61 ± 0.14	1.45 ± 0.55
rNbVCAM1-His_6_-alkyne (CuAAC chemistry)	2.10 ± 0.71	8.67 ± 0.27	N/D
NbVCAM1-His_6_ (EDC/NHS chemistry)	20.70 *	49.30 *	N/D

N/D: not determined, *: insufficient data to calculate the standard error.

## Supplementary Materials

Supporting information associated to this research (expression, purification and characterization of nanobodies, regeneration of nanobody-conjugated surfaces, as well as SPR kinetics data) are available online at www.mdpi.com/2079-6374/6/3/34/s1.
