# Supplementary material for: Enhanced Biosensor Platforms for Detecting the Atherosclerotic Biomarker VCAM1 Based on Bioconjugation with Uniformly Oriented VCAM1-Targeting Nanobodies
Source: Biosensors (Basel). 2016 Jul 5;6(3):34. doi: 10.3390/bios6030034 (PMC5039653; doi:10.3390/bios6030034)
Supplement: Supplementary file 1 [file biosensors-06-00034-s001.pdf]

Supplementary Materials: Enhanced Biosensor Platforms for Detecting the Atherosclerotic Biomarker VCAM1 Based on Bioconjugation with Uniformly Oriented VCAM1-Targeting Nanobodies

Duy Tien Ta, Wanda Guedens, Tom Vranken, Katrijn Vanschoenbeek, Erik Steen Redeker, Luc Michiels and Peter Adriaensens

Protein Expression and Purification

The expression plasmid pMXB10:NbVCAM1-LEY-intein-CBD was transformed into *E. coli* SHuffle® T7 cells which were then selected on LB-agar plate supplemented with 100 µg/mL Ampicillin (LB^{Amp}). A fresh single colony was then selected and pre-cultured in 3 mL LB^{Amp} at 37 °C overnight while shaking. On the next day, the pre-culture was inoculated into 300 mL LB^{Amp} and the cells were grown at 37 °C while shaking until the OD_{600} reached 0.5. Protein expression was then induced by adding Isopropyl β-D-1-thiogalactopyranoside (IPTG) at a final concentration of 0.5 mM and the cells were cultured at 37 °C for 3 more h. Finally, the cells were harvested by centrifuging at 5000 g for 10 min.

Cytoplasmic extraction was performed by resuspending the cells (of a 300 mL culture) in 6 mL B-PER reagent (supplemented with 6 units of *DNase*I) and incubating at room temperature for 15 min. Clear cell lysate was obtained by centrifuging at 20,000 *g* for 30 min at 4 °C. The total cell protein content before and after IPTG induction, as well as the cell lysate and the cell debris were analyzed on a 15% SDS-PAGE gel. On the other hand, the expression and purification of the NbVCAM1-His_6 was carried out as described by Saerens, et al. (2004). This nanobody was used as positive controls in the ELISA.

The clear cell lysate from cells expressing the NbVCAM1-LEY-intein-CBD was loaded on 5 mL, 5 mm-diameter columns packed with 1.5 mL chitin resin, pre-equilibrated with column buffer (CB) (20 mM HEPES, 500 mM NaCl and 1 mM EDTA at pH 8.5). The columns were thoroughly washed with 20 bed volumes of CB. Cleavage of the nanobody from the intein was done by quickly flushing each column with 1.5–2 bed volumes and then filling with 1 bed volume of: (i) CB containing 30 mM DTT (to produce the NbVCAM1-LEY); or (ii) CB containing 1 mM cysteine-alkyne linker supplemented with 30 mM 2-mercaptoethane sulfonic acid sodium salt (MESNA) and 1 mM Tris(2-carboxyethyl)phosphine (TCEP) (to produce the NbVCAM1-LEY-alkyne. The columns were incubated at 4 °C overnight after which the proteins were eluted with 2 bed volumes of column buffer. The eluates were analyzed on a 15% SDS-PAGE gel to investigate the EPL-mediated cleavage efficiency, and were desalted and buffer-exchanged with phosphate buffer saline (PBS, 137 mM NaCl, 2.7 mM KCl, 10 mM Na_2HPO_4 and 2 mM KH_2PO_4 at pH 7.4) using the Amicon® Ultra concentrator (MWCO 3000, Merck Chemicals N.V./S.A., Belgium). All buffers were filtered through a 0.22 µm membrane and aerated with nitrogen gas before use. The NbBcII-10-LEY-alkyne nanobody was produce using the similar protocol and was used as negative control in ELISA and SPR. All the protein concentrations were determined using the BCA protein assay kit.

Electrospray Ionization—Fourier Transform Mass Spectrometry (ESI-FTMS)

The desalted nanobodies were subjected to HPLC-MS to confirm their masses. Using a Dionex 3000 HPLC and auto-injector configuration, 30 µL aliquots of protein solutions were trapped and desalted for 5 min on a Dionex Acclaim PolarAdvantage II C18 reversed-phase 2.0 × 10 mm guard column (particle diameter 5 µm, porosity 120 Å) at a flow rate of 0.5 mL·min^{-1} using 0.1% *v*/*v* formic acid in Milli-Q water (solvent A) directed to waste. Upon valve switching, elution was started for 7 min pumping 50% *v*/*v* acetonitrile in solvent A at a reduced flow rate of 0.1 mL·min^{-1} into an electrospray ionization source with heated auxiliary gas (5 arbitrary flow rate units; 55 °C). Source voltage and capillary temperature was +4 kV and 275 °C, respectively. The Orbitrap Velos Pro FTMS

(Thermo Scientific, Aalst, Belgium) controlled by Thermo Xcalibur software v2.2 was operated in full scan mode in the mass range 110–2000 Thomson at a resolution of 3 × 104 FWHM with automatic gain control set to 1 × 106 ions in maximal 100 msec, without microscan averaging. Scans recorded between elution time 4 and 7 min were averaged. For molecular weight determination the average spectrum was deconvoluted using Promass software for Xcalibur v2.8 (Novatia LLC). The deconvoluted masses were then compared with the theoretical values which were determined using CLC Main Workbench 6 software. Horse heart cytochrome C (8 μM in solvent A) was infused by a syringe pump at 5 $\mu L{\cdot}min^{-1}$ as a tuning and calibration standard (average molecular mass of 12,360 Da).

Enzyme-Linked Immuno-Sorbant Assay (ELISA)

To determine the antigen binding capacity of the different nanobody variants towards recombinant human VCAM1 (hVCAM1) from R & D Systems, the nanobodies were subjected to a sandwich ELISA. The NbVCAM1-His_6 and NbBcII-10-His_6 were used as positive and negative controls, respectively. A 96-well microplate was coated with 200 μL of a 5 μM nanobody solution in 0.1 M $NaHCO_3$ pH 8.2. After incubation overnight at 4 °C, the plate was washed five times with Tris-buffered saline pH 8.0 (TBS) containing 0.5% (*v*/*v*) Tween 20 (TBST), blocked with 200 μL of 5% (*w*/*v*) skim milk powder in TBST at room temperature for 2 h and washed again five times with TBST. To each well, 100 μL of recombinant hVCAM1 in TBST was added in a concentration range of 0–100 ng/mL. The plate was incubated at room temperature for 5 h before washing five times with TBST. The captured antigen was incubated with 100 μL of 1 μg/mL mouse monoclonal anti-human IgG-alkaline phosphatase antibody (in TBST) at room temperature for 2 h, followed by washing three times with TBST. Subsequently, 200 μL of *p*-nitrophenyl phosphate (PNPP) substrate was added to each well and the plate was incubated at 37 °C for 30 min before adding 50 μL of 3 M NaOH to stop the reaction. The plate was read immediately with a FLUOStar Omega Reader (BMG Labtech) to measure the absorbance at 405 nm (OD_{405}). All measurements were performed in triplicate and the data were processed with Graphpad Prism 5.0 software for statistical analysis.

CuAAC 'Click' Reaction with Azido-Biotin

The 'click' reactions was carried out in 200 μL PBS containing 10 μM purified nanobody, 0.2 mM azido-biotin, 1 mM TCEP, 0.1 mM tris-(Benzyltriazolylmethyl)amine (TBTA) and 1 mM $CuSO_4$. The 'click' reaction was performed at room temperature under shaking for 2 h. The biotinylated product was analyzed by Western blotting as follows: after analysis on a 15% SDS-PAGE gel, the proteins were transferred to an Amersham Hybond™-LFP PVDF membrane. The blot was blocked with 5% BSA for 1.5 h to inhibit non-specific binding. The biotinylated protein was incubated with streptavidin-alkaline phosphatase conjugate for 1 h and visualized by incubation with nitro blue tetrazolium chloride/5-bromo-4-chloro-3-indolyl phosphate (NBT/BCIP) substrate.

Table S1. Efficiency of the CuAAC-mediated coupling of NbVCAM1-LEY-alkyne to azidified silicon wafers in different buffers as detected by ellipsometry. The efficiency is expressed in surface mass density (ng protein per cm^2) as calculated using the simplified Lorentz-Lorenz relation.

Buffer	PBS pH 7.4	Acetate pH 4.0	Acetate pH 5.0	Acetate pH 6.0
Surface mass density (ng/cm^2)	166 ± 4	229 ± 1	135 ± 1	160 ± 1

Table S2. Comparison of the hVCAM1 (R & D Systems) antigen binding capacity of the nanobody-conjugated silicon wafers (surface mass density in ng/cm^2) after regeneration with 10 mM Glycine-HCl pH 2.5 and 0.5% SDS solution, as measured by ellipsometry.

	1st Binding Cycle (ng/cm^2)	2nd Binding Cycle (ng/cm^2)	% Loss of Binding Capacity
NbVCAM1-LEY-alkyne	532	444	16.6%
rNbVCAM1-His_6-alkyne	436	408	6.4%

Table S3. Binding kinetic data obtained for the human and mouse recombinant VCAM1 antigen binding in PBS to the nanobody conjugated flow cells prepared by the different coupling methods. The k_a (association rate constant in $M^{-1}\cdot s^{-1}$) and k_d (dissociation rate constant in s^{-1}) were determined using the single-cycle kinetics module. Data obtained from three replicates. (N/A: not available due to the inability to calculate the kinetic parameter with statistical significance. N/D: not determined.)

k_a	hVCAM1 (R & D System)			hVCAM1 (Peprotech)		mVCAM1 (Bioconnect)		
NbVCAM1-LEY-alkyne (CuAAC chemistry)	3.01E + 05	1.26E + 05	7.82E + 04	1.76E + 06	3.44E + 06	2.92E + 05	6.44E + 04	7.94E + 04
rNbVCAM1-His6-alkyne (CuAAC chemistry)	1.39E + 04	1.84E + 04	34170	48050	41110	N/D		
NbVCAM1-His6 (EDC/NHS chemistry)	5.07E + 03	N/A	9.82E + 03	N/A	N/A	N/D		
**k_d**	**hVCAM1 (R & D System)**			**hVCAM1 (Peprotech)**		**mVCAM1 (Bioconnect)**		
NbVCAM1-LEY-alkyne (CuAAC chemistry)	4.88E–05	1.46E − 05	9.81E − 05	3.13E − 03	6.15E − 03	8.66E − 05	9.16E − 05	2.09E − 04
rNbVCAM1-His6-alkyne (CuAAC chemistry)	5.10E − 05	3.57E − 05	3.17E − 04	3.91E − 04	3.54E − 04	N/D		
NbVCAM1-His6 (EDC/NHS chemistry)	1.05E − 04	N/A	4.84E − 04	N/A	N/A	N/D		

Table S4. Comparison of the hVCAM1 (R & D Systems) antigen binding capacity (expressed as RU_{max}) of the conjugated NbCAM1-LEY-alkyne during 3 binding cycles as measured by SPR. Hereto, the flow cell surface was first azidified and then conjugated with NbVCAM1-LEY-alkyne via CuAAC in acetate buffer pH 4.0. After completion of the first antigen binding cycle as described in the Materials and Methods section, regeneration took place by rinsing with 0.5% SDS solution. After the second antigen binding cycle, regeneration was accomplished by means of 10 mM NaOH. Finally, the third binding cycle was carried out and the loss in binding capacity was evaluated for each regeneration step.

1st Binding Cycle (RU_{max})	2nd Binding Cycle (RU_{max})	3rd Binding Cycle (RU_{max})	% Loss in Binding Capacity (2 vs. 1)	% Loss in Binding Capacity (3 vs. 2)
42.6	28.7	27.6	32.6%	3.9%

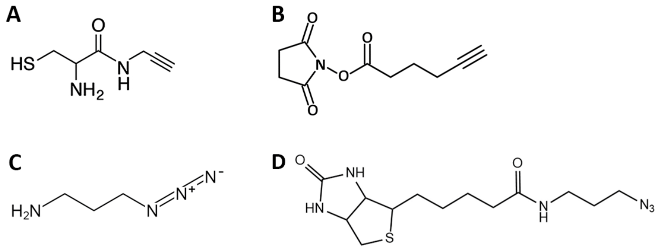

Figure S1. Chemical structure of the linkers used: (**A**) Cysteine-alkyne; (**B**) *N*-hydroxysuccinimide (NHS) derived ester linker 2,5-dioxopyrrolidin-1-ylhex-5-ynoate; (**C**) azido-propylamine; and (**D**) azido-biotin.

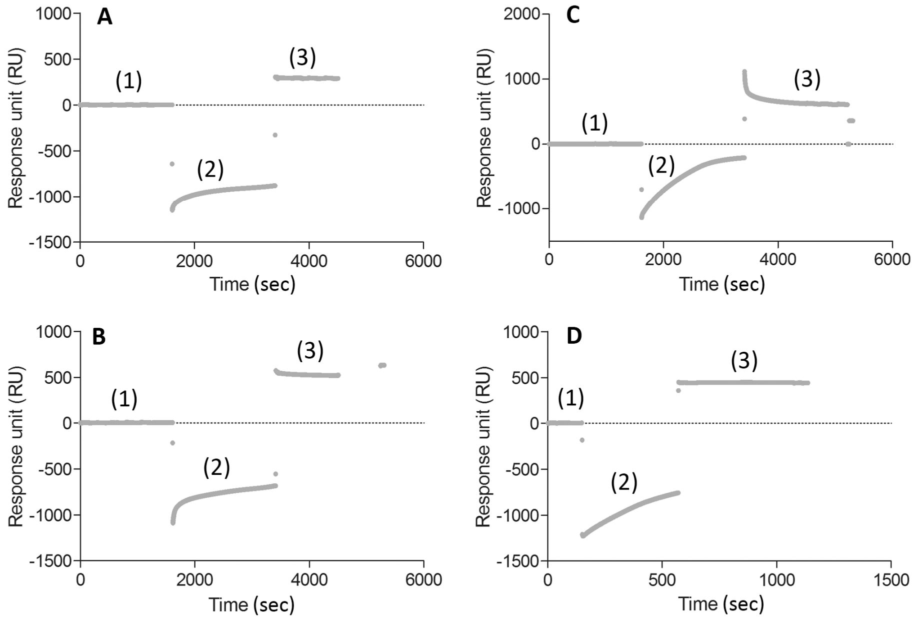

Figure S2. Conjugation of a SPR C1 Series S chip with (**A**) NbBcII-10-LEY-alkyne (Fc1); (**B**) NbVCAM1-LEY-alkyne (Fc2); (**C**) rNbVCAM1-His_6-alkyne (Fc3) and (**D**) NbVCAM1-His_6 (Fc4) as measured on the Biacore™ T200 System. The nanobody conjugations on Fc1, 2 and 3 were performed via CuAAC 'click' chemistry in acetate buffer pH 4.0, whereas conjugation on Fc4 was carried out using EDC/NHS coupling. Time events: (1) baseline calibration in PBS; (2) conjugation with nanobodies; (3) washing with PBS.

Reference

1. Saerens, D.; Kinne, J.; Bosmans, E.; Wernery, U.; Muyldermans, S.; Conrath, K. Single domain antibodies derived from dromedary lymph node and peripheral blood lymphocytes sensing conformational variants of prostate-specific antigen. *J. Biol. Chem.* **2004**, *279*, 51965–51972.
